# Accelerated retinal ganglion cell death in mice deficient in the Sigma-1 receptor

**Published:** 2011-04-26

**Authors:** Timur A. Mavlyutov, Robert W. Nickells, Lian-Wang Guo

**Affiliations:** 1 Department of Pharmacology, University of Wisconsin, School of Medicine and Public Health, Madison, WI; 2 Department of Ophthalmology and Visual Sciences, University of Wisconsin, School of Medicine and Public Health, Madison, WI

## Abstract

**Purpose:**

The sigma-1 receptor (σR1), a ligand-operated chaperone, has been inferred to be neuroprotective in previous studies using σR1 ligands. The σR1 specificity of the protective function, however, has yet to be firmly established, due to the existence of non-σR1 targets of the ligands. Here, we used the σR1-knockout mouse (*Sigmar1^−/−^*) to demonstrate unambiguously the role of the σR1 in protecting the retinal ganglion cells against degeneration after acute damage to the optic nerve.

**Methods:**

Retinal σR binding sites were labeled with radioiodinated σR ligands and analyzed by autoradiography. Localization of the σR1 was performed by indirect immunofluorescence on frozen retinal sections. Retinal ganglion cell death was induced by acute optic nerve crush in wild-type and *Sigmar1^−/−^* mice. Surviving cells in the ganglion cell layer were counted on Nissl-stained retinal whole mounts 7 days after the crush surgery.

**Results:**

Photoaffinity labeling indicated the presence of the σR1 in the retina, in concentrations equivalent to those in liver tissue. Immunolabeling detected this receptor in cells of both the ganglion cell layer and the photoreceptor cell layer in wild-type retinas. Quantification of cells remaining after optic nerve crush showed that 86.8±7.9% cells remained in the wild-type ganglion cell layer, but only 68.3±3.4% survived in the *Sigmar1^−/−^*, demonstrating a significant difference between the wild-type and the *Sigmar1^−/−^* in crush-induced ganglion cell loss.

**Conclusions:**

Our data indicated faster retinal ganglion cell death in *Sigmar1^−/−^* than in wild-type mice under the stresses caused by optic nerve crush, providing direct evidence for a role of the σR1 in alleviating retinal degeneration. This conclusion is consistent with the previous pharmacological studies using σR1 agonists. Thus, our study supports the idea that the σR1 is a promising therapeutic target for neurodegenerative retinal diseases, such as glaucoma.

## Introduction

The sigma-1 receptor (σR1), a membrane protein of 26.2 kDa [1], represents a unique drug-binding site that has no homology to any other known mammalian proteins [2]. It is widely distributed in the central nervous system, including the eye [3-6]. The sequence of the σR1 is highly conserved across mammalian species, implicating fundamental biologic function(s) [2]. The sigma-2 receptor (σR2) subtype has been identified pharmacologically [7], but has yet to be cloned.

Although the σR1 signaling pathway(s) remain unclear, it has been discovered that the σR1 is a Ca^2+^-sensitive and ligand-operated chaperone primarily residing in the mitochondria-associated endoplasmic reticulum (ER) membrane [8]. Upon ER Ca^2+^ depletion caused by cellular stresses, the σR1 dissociates from the binding immunoglobulin protein (BiP; another ER chaperone), and becomes available to regulate inositol trisphosphate (IP3) receptor-mediated Ca^2+^ release to maintain mitochondrial Ca^2+^ homeostasis. The σR1 is therefore protective against apoptosis. Under prolonged cellular stresses, the σR1 translocates to the extended ER network, whereby it interacts with and regulates the function of a variety of ion channels, receptors, or kinases. Thus, the σR1 is proposed to function as an interorganelle-signaling modulator [2].

Recently, a possible neuroprotective function of the σR1 has attracted growing interest. Some σR1 agonists have been shown to attenuate neuronal loss in the brain upon acute neurodegeneration [9,10], and also to promote neurite outgrowth of PC12 cells [11] and motoneurons [12]. The σR1 ligand-activated protective effects have also been explored in the mouse and rat retinas, where the presence of the *σR1* mRNA and its expression have been reported [4,5,13,14]. The σR1 ligands dehydroepiandrosterone-sulfate (DHEA-S) and PRE-084 attenuated retinal damage in rats [15,16]. Another σR1 agonist, (+)-pentazocine, reduced glutamate-initiated cell death, in both cultured primary ganglion cells [17] and RGC-5 cells [18,19]. When injected intraperitoneally into the diabetic mice, (+)-pentazocine reduced retinal lipid peroxidation and cell loss in the ganglion cell layer [20]. These reports shed light on the σR1 as a potential target for new therapeutic agents to treat retinal neurodegeneration.

A variety of small molecules are known to bind the σR1, and some of them have been used for pharmacological interventions of disease states such as depression (for reviews, see [21,22]). However, it is known that the σR1 ligands can also bind to other receptors. For instance, even the highly σR1-selective ligands (+)-pentazocine and (+)-SKF-10047 have alternative targets [22]. N,N-dimethyl tryptamine (DMT), which has been recently identified as an endogenous ligand for the σR1 [23], is a more potent agonist for serotonin receptors [24]. This complexity of drug-target interactions often confounds the specificity and underlying mechanisms of cellular or physiologic responses elicited by σR1 ligands. It is thus important to define a σR1-specific protective function. A direct approach to address this issue is to examine retinal neurodegeneration in vivo in the *Sigmar1^−/−^* mouse [25]. Although *Sigmar1^−/−^* mice do not show overt phenotypes [25], under certain stress conditions, significant differences in motor activities between the *Sigmar1^−/−^* and wild-type (WT) have been observed [6,23].

In this study, we have generated stresses for ganglion cells in *Sigmar1^−/−^* and WT mice by applying optic nerve crush, which is an established model for acutely inducing an apoptotic program similar to the one executed in glaucomatous retinal ganglion cells [26-28]. Comparison of cell loss in the ganglion cell layer of *Sigmar1^−/−^* and WT mice revealed a greater degree of ganglion cell death in the absence of the σR1, demonstrating the σR1-specific protection against cell death. Moreover, a high abundance of the σR1 in the retina, visualized by photoaffinity labeling and immunostaining, also supports the σR1 as a potential target for treating neurodegenerative retinal diseases.

## Methods

### Source of animals

*Oprs1* mutant (+/−) *Oprs^Gt(IRESBetageo)33Lex^* litters on a C57BL/6J×129s/SvEv mixed background were purchased from the Mutant Mouse Regional Resource Center, UC Davis, CA, from which homozygous wild-type (*Sigmar1^+/+^*) and σR1 knockout (*Sigmar1^−/−^*) mice were obtained through in-house breeding [6]. The genotypes were confirmed by PCR. The primers for *Sigmar1^+/+^*: TCT GAG TAC GTG CTG CTC TTC G and CAG AAA TCT CAG CCC AGT ATC G. The primers for *Sigmar1*^−/−^: TCT GAG TAC GTG CTG CTC TTC G and ATA AAC CCT CTT GCA GTT GCA TC. All mice were maintained on a 4% fat diet (8604 M/R, Harland Teklad, Madison, WI) and subjected to standard light cycles (12 h:12 h light-dark). The animals were handled in accordance with animal care and use guidelines of the University of Wisconsin, Madison, WI and in compliance with the ARVO Statement for the Use of Animals in Ophthalmic and Vision Research.

### Preparation of retinal homogenates

Mice were euthanized by CO_2_ asphyxiation followed by cervical dislocation, and eyes were enucleated immediately. Retinas were carefully dissected to prevent contamination of the retinal pigment epithelium (RPE) cell layer. The retinas were minced and then homogenized on ice with a glass homogenizer (Teflon pestle by six slow passes at 3,000 rpm) in PBS buffer (10 mM Na_2_HPO_4_/1.76 mM KH_2_PO_4_, pH 7.4, 137 mM NaCl, 2.68 mM KCl) containing the Complete Protease Inhibitor Cocktail (Roche, Indianapolis, IN). Homogenized tissues were then centrifuged at 15,000× g for 10 s to remove cell debris. The membrane suspension in the supernatant was snap-frozen with dry ice/ethanol, and stored at −80 °C at a final protein concentration of 20 mg/ml. Bovine retinal homogenates [29] and rat liver membranes [30] were prepared as previously described. Protein concentrations of the retinal and liver homogenates were determined by the Lowry protein assay.

### Photoaffinity labeling

Radiochemical synthesis of the sigma receptor photolabel [^125^I]-IAF (1-N-(2’,6’-dimethyl-morpholino)-3-(4-azido-3-[^125^I]iodo-phenyl propane) [30] and [^125^I]-IACoc (3-iodo-4-azido cocaine) [31] was performed as previously described. For photoaffinity labeling, retinal homogenates were incubated in the presence and absence of 5 μM (+)- pentazocine in 60 mM Tris, pH 7.4, for 25 min at 22 °C, then [^125^I]-IAF or [^125^I]-IACoc was added to a concentration of 1 nM and incubated for another 15 min. Samples were irradiated for 6 s with a high-pressure AH6 mercury lamp (AH6-IC-30222; Advanced Radiation Corpotation, Santa Clara, CA), and the reaction was immediately quenched with the sample buffer containing 250 mM β-mercaptoethanol. Proteins were separated on a 16.5% sodium dodecyl sulfate (SDS) gel (18 cm×16 cm), and photolabeling was detected by PhosphorImager (445 SI; Molecular dynamics, Sunnyvale, CA).

### Immunohistochemistry

Following euthanasia of the mice, their eyes were enucleated immediately and dissected. The eyecups were fixed in 4% paraformaldehyde for 7 h, and then cryoprotected in 30% sucrose in PBS for another 14 h, all at 4 °C. Cryosections of 10 μm each were cut from the eyecups frozen in the optimum cutting temperature (O.C.T.) embedding medium (Sakura Finetek 4583, Sakura Finetek USA, Inc., Torrance, CA), and used for immunostaining according to the method described previously [31] with minor modifications. Briefly, retinal sections were permeabilized with 1% Triton X-100 in PBS for 20 min, blocked with 10% normal goat serum (Cat#71–00–27; Kirkegaard & Perry Laboratories, Gaithersburg, MD) for 2 h at 22 °C, and then incubated with purified rabbit anti-sigma-1 receptor antibody (1/150 dilution) [32] and mouse monoclonal anti-synaptophysin (Cat. #MAB368, 1/500 dilution; Chemicon, Los Angeles, CA) overnight at 4 °C. After rinsing the sections 3×, secondary antibodies (Alexa-488 conjugated goat-antirabbit and Alexa-594-conjugated goat-antimouse) at 2 μg/ml was applied at 22 °C for 2 h. Sections were then rinsed 3×, counterstained with 4',6-diamidino-2-phenylindole (DAPI) for 5 min, and mounted in the Prolong Gold mounting medium (Invitrogen, Carlsbad, CA) and coverslipped. The slides were left in the dark overnight and then sealed using clear nail polish (Electron Microscopy Sciences, Hatfield, PA). Microscopy of the retinal sections was first performed on a Zeiss Axiovert 200 M epifluorescent microscope equipped with a 100× oil objective and the Axiovision 4.3 software (Carl Zeiss Light Microscopy, Göttingen, Germany). Images were then taken with a Nikon A1R laser confocal microscope (Nikon, Tokyo, Japan) supplied with a green 488 nm Argon laser and a red 561 nm DPSS laser through an Apo60X VC oil-immersion objective with NIS elements software [6]. Z-stacks were collected at 0.5 μm each, for a total thickness of 15 μm. Sequentially collected images were stacked together in the ImageJ program using the standard deviation option. Final figures were made in Adobe Illustrator (Adobe Systems Inc., San Jose, CA).

### Optic nerve crush and ganglion cell counting

The intraorbital optic nerve crush surgery was conducted as described in detail previously [27]. Experiments were performed using five age-matched pairs (WT and *Sigmar1^−/−^*) of 6- and 12-month-old mice. Prior to the crush surgery, mice were anesthetized by intraperitoneal injection of 0.2 ml PBS solution containing ketamine (6 mg/ml) and xylazine (0.4 mg/ml) [27]. In each mouse, the left eye underwent surgery, while the right eye served as an untreated control.

Cell loss was quantified from Nissl-stained images of retinal wholemounts as described previously [28] and is expressed as a percentage of cells present in experimental retinas relative to the untreated fellow retinas of the same mice. Mice were euthanized, and the superior region of each eye was marked with an ophthalmic cautery. The eye was enucleated and fixed in 4% paraformaldehyde in PBS buffer for 1 h at 22 °C. After rinsing the eyeball in PBS, the cornea and lens were removed, the eyecup was incubated in PBS containing 0.3% TritonX-100 (v/v) overnight at 22 °C. The retina was then isolated, mounted with the ganglion cell layer up on a glass “Plus” slide (Fisher Scientific, Chicago, IL), and flattened under a coverslip with a 10 g weight on the top. Air-dried retinas were stained with 1% cresyl violet acetate (Nissl stain, in 0.25% acetic acid) by brush-painting, differentiated and dehydrated in 100% ethanol, cleared in xylene, and finally mounted in the Prolong Gold mounting medium and coverslipped.

We then counted cell numbers in the ganglion cell layer using these retinal whole-mount slides. Digital images were taken at 200× magnification using an Olympus BX40 light microscope (Olympus, Mellville, NY) with a SONY DXC-390 video camera attachment (Sony, New York, NY), and imported into Image Pro Plus v4.5 (Media Cybernetics, Inc., Silver Spring, MD) quantification software. Images were taken in at least four microscopic fields (each encompassing an area of 0.33 mm^2^) in the peripheral to midperipheral region around the four quadrants of each retina. On each image, five representative areas were chosen, and the cells in each area were counted. The remaining cells in each treated eye were then calculated as a percentage of the corresponding counts of the untreated control.

Overall, approximately 10% of the cells present in each retinal whole-mount were counted, providing an accurate estimate of the loss of retinal ganglion cells after optic nerve crush, as demonstrated by previous studies [27,28,33].

## Results

### Sigma receptor binding sites in the retina detected by photoaffinity labeling

While ligands have been frequently used to target the σR1 in mouse and rat retinas in previous studies [15,16,20,32,34], the ligand-binding properties of the σR1 (and σR2) have not been thoroughly examined. Here, we explored the retinal σR binding sites using radio-iodinated photoreactive σR ligands [^125^I]-IACoc and [^125^I]-IAF. These two photoaffinity probes have often been used to specifically photolabel sigma receptors. [^125^I]-IACoc labels only the σR1, whereas [^125^I]-IAF labels both the σR1 and the σR2 [23,30,35,36]. The specificity of these probes for the σR1 has been exhibited by the lack of specific photolabeling either in the *Sigmar1^−/−^* samples or in the presence of (+)-pentazocine, a potent σR1 ligand [23]. Specific labeling of the σR2 by [^125^I]-IAF has been demonstrated by the protection of haloperidol, which binds both the σR1 and the σR2 [23,30].

**Figure 1:**
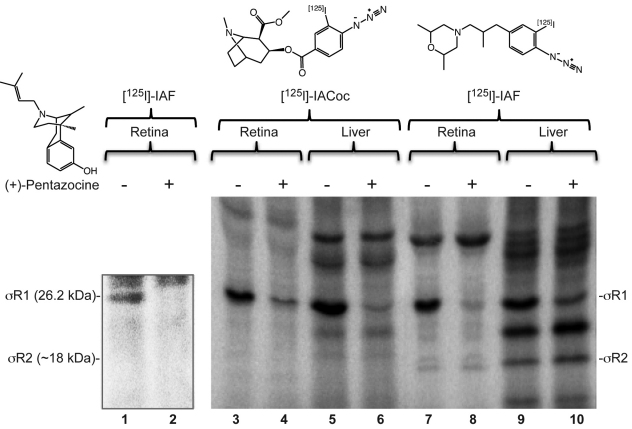
Photoaffinity labeling of the sigma-1 receptor in the retina. Shown are autoradiograms of the photolabeled retinal homogenates that were resolved on sodium dodecyl sulfate gels. Photoaffinity labeling of σRs in the mouse retinal homogenates was performed with [^125^I]-IAF (lanes 1 and 2). In bovine retinal homogenates, [^125^I]-IACoc (lanes 3 and 4) and [1^25^I]-IAF (lanes 7 and 8) were used, and labeling was compared to that in rat liver membranes (lanes 5 and 6, 9 and 10, respectively). The specificity of the σR1 photolabeling was demonstrated by the reduced intensity in the presence of the σR1 ligand (+)-pentazocine (the even-numbered lanes). In contrast, the nonspecifically labeled upper bands were not affected by (+)-pentazocine. The typical labeling pattern of the σR1/σR2 bands (seen in lane 9) have often been observed previously in mouse and rat liver membranes [23,30]. In each lane of the gel, 200 μg of total protein was loaded.

As shown in Figure 1, [^125^I]-IAF specifically photolabeled the σR1 (the 26 kDa band) in mouse retinal homogenates (lane 1), because the labeling was blocked by the presence of the σR1-specific ligand (+)-pentazocine (lane 2). Since bovine retinal homogenates can be readily prepared to provide adequate samples for biochemical determinations, we also used bovine samples to analyze the σR binding sites in mammalian retinas. Similar to the results obtained from the mouse retina, the σR1 was detected specifically by [^125^I]-IAF (lanes 7 and 8) and [^125^I]-IACoc (lanes 3 and 4). The intensity of the σR1 labeling in the retina (lanes 3 and 7) was comparable to that in the liver (lanes 5 and 9), which is known to be rich in the σR1 and is often used for σR ligand-binding studies [23,30,37].

Interestingly, unlike in the rat liver (lane 9), very low (if any) σR2 was detected in either the bovine (lane 7) or mouse (lane 1) retina. Since the σR2 has not been cloned, photoaffinity labeling represents a sensitive approach for visualizing this subtype. However, considering that [^125^I]-IAF labels the σR1 more efficiently than does the σR2 [30], this data did not preclude the possibility of a σR2 presence that might have been undetectable. More experiments are underway to address this question.

### High abundance of the sigma-1 receptor in the retinal ganglion cells demonstrated by immunostaining

Previous immunohistochemical studies have led to contradictory results with regard to the presence of the σR1 in various types of retinal neurons. For example, an earlier study showed the σR1 presence in the photoreceptor nuclear region and the inner segment [4], but in a more recent report, the σR1 was not found in rat photoreceptor cells [5]. This discrepancy may have arisen from technical issues such as sources of the σR1 antibodies, levels of background, et cetera. Here, we clarified this using an antibody prepared against the full-length purified σR1 protein that retained full [^3^H]-(+)-pentazocine binding capacity [38]. Importantly, using the *Sigmar1*^−/−^ mouse as a negative control, this σR1 antibody proved to be highly specific for immunostaining the σR1 in the mouse spinal cord [6].

**Figure 2:**
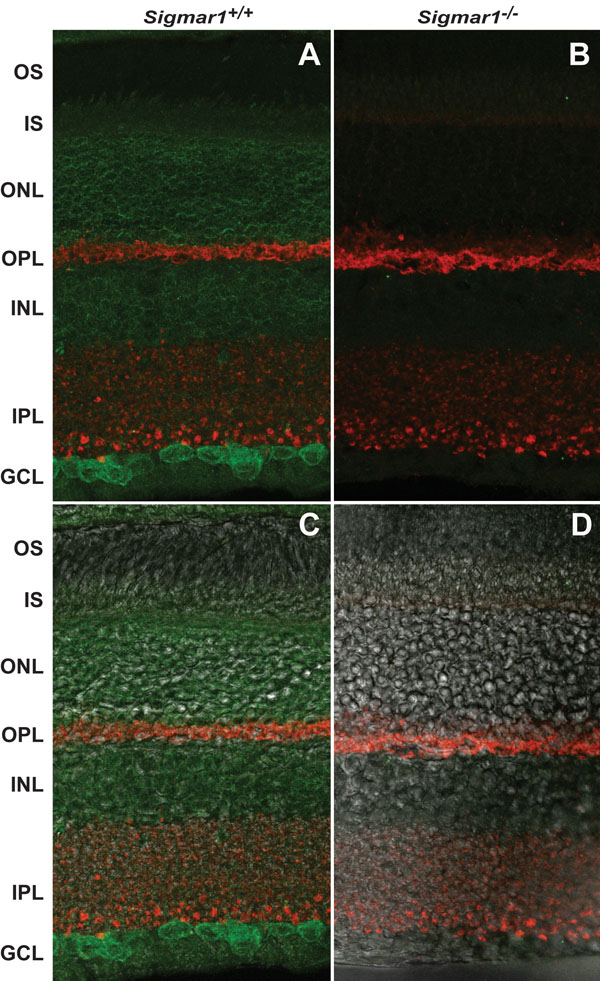
The sigma-1 receptor distribution in the mouse retina. **A**: Immunostaining of the σR1 (green) in the WT mouse retina. **B**: Immunostaining of the σR1 in the *Sigmar1*^−/−^ mouse retina (negative control). **C** and **D**: The Nomarsky image superimposed with the staining images in **A** and **B**, respectively. The abbreviations of the distinct cell layers are: OS, outer segment; IS, inner segment; ONL, outer nuclear layer; OPL, outer plexiform layer; INL, inner nuclear layer, IPL, inner plexiform layer; GCL, ganglion cell layer. Synaptophysin was stained (red) to mark the presynaptic terminals in the OPL and IPL. Immunostaining of the σR1 was performed on the mouse retinal cryosections using the antibody raised against the full-length σR1 [6,32], followed by incubation with the Alexa-488 conjugated goat-antirabbit antibody. WT and *Sigmar1^−/−^* mice of the same age (3 months) were used for preparation of retinal sections. Images were taken on a Nikon A1R laser confocal microscope and processed using Adobe Photoshop.

Figure 2 shows the side-by-side comparison of the σR1 immunostaining in WT and *Sigmar1*^−/−^ retinas; both experiments were performed under the same conditions. The specificity of the σR1 staining in the WT retina (Figure 2A,C) was manifested by the lack of green fluorescence from the *Sigmar1^−/−^* control (Figure 2B,D). In agreement with previous reports [4,5], the σR1 was highly expressed in the mouse ganglion cell layer. Remarkably, the outer nuclear layer, which contains the photoreceptor nuclei, was also intensely stained for the σR1. The staining was found mostly around the nuclei. This result was consistent with an early report of mRNA presence and expression of the σR1 in the mouse outer nuclear layer [4,13], but disagreed with a recent report that concluded the absence of the σR1 in rat photoreceptor cells [5]. Immunostaining of the σR1 was also found in the photoreceptor inner segment, albeit with a low intensity, but not in the outer segment. Consistently, the σR1 has been identified to be a molecular chaperone primarily residing in the ER membrane [8], and has been typically immunolocalized in the ER network around the nucleus in Chinese hamster ovary cells [8], retinal ganglion cells [4], and Müller cells [39]. The photoreceptor outer segment, a highly specialized compartment, does not contain an ER [31]. No or very low level of σR1 was detected in the photoreceptor synaptic terminals in the outer plexiform layer, which was marked by the staining of synaptophysin (red, Figure 2A).

Thus, our data demonstrated the σR1 expression in ganglion cells, as well as in photoreceptor cells. Our high confidence in this data are based on the absence of σR1 staining in the *Sigmar1^−/−^* retina.

### The ganglion cells in *Sigmar1^−/−^* mice are more susceptible to optic nerve crush

To compare the susceptibility of WT and *Sigmar1^−/−^* retinal ganglion cells to neurodegeneration, we used the optic nerve crush surgery to induce acute ganglion cell death [27]. The loss of ganglion cells after the surgery is a continuous apoptotic process over an approximately three-week period [28]. In consideration of possibly faster cell death in *Sigmar1*^−/−^ mice, we chose a time point of one week after the crush surgery to examine cell loss in the ganglion cell layer, in both WT and *Sigmar1^−/−^* retinas.

**Figure 3:**
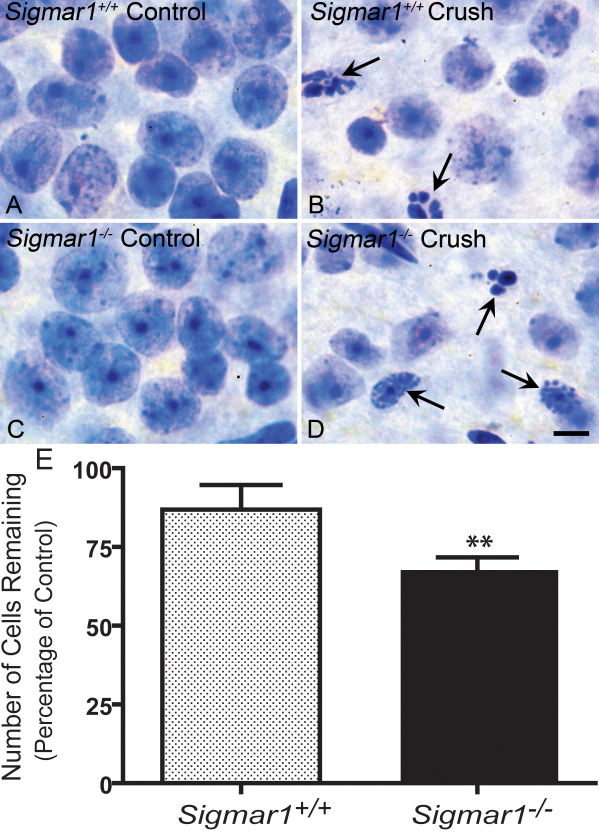
Comparison of the post-crush cell loss in the retinal ganglion cell layer between WT and *Sigmar1^−/−^* mice. **A**-**D**: Nissl-stained retinal whole-mounts from WT (**A** and **B**) and *Sigmar1^−/−^* (**C** and **D**) mice. Images were from representative fields (1,000×) of the mid-peripheral inferior retinas of 12-month-old mice. For each mouse, while the right eye served as untreated control (**A** and **C**), the left eye was treated by optic nerve crush for 3 s (**B** and **D**). Retinal whole-mounts were prepared 7 days after surgery, and the side of the ganglion cell layer was stained. Healthy ganglion cells exhibited larger somas and nuclei with prominent nucleoli. Arrows point to apoptotic cells. **E**: Quantification of cells remaining in the retinal ganglion cell layer one week after surgery. The number of remaining cells in the experimental eye is represented as a percentage of the untreated control. The data were pooled from three WT and *Sigmar1^−/−^* pairs of 6-month-old mice and two pairs of 12-month-old mice. There were 86.82±7.90% (mean±standard deviation [SD], n=5) cells remaining in WT mice and 68.31±3.36% remaining in *Sigmar1^−/−^* mice. ** *t*-test, p=0.0013.

Degenerating cells appeared 7 days after the crush surgery, as characterized by the dense, fragmented nuclei staining (Figure 3B,D). In contrast, in the untreated control eyes, the nuclei in the ganglion cell layer showed an intact and round healthy appearance (Figure 3A,C).

We then counted the remaining healthy cells on retinal whole mounts. The densely stained fragmented nuclei (dying cells) and spindle-shaped nuclei (vascular endothelial cells) were excluded. The selection did not distinguish between ganglion cells and large amacrine cells, but previous studies have estimated the proportion of ganglion cells in this layer to be 40%–60% of the neurons present [27,40,41].

There was no difference in cell density in the control eyes of the 12-month-old WT (34.02 cells/100 μm^2^) and *Sigmar1^−/−^* mice (34.41 cells/100 μm^2^), indicating normal development in both the WT and *Sigmar1^−/−^* ganglion cell layers (also see Figure 3A,C).

In response to optic nerve crush, however, significantly more cells were lost in *Sigmar1^−/−^* mice one week after surgery. Data pooled from 6- and 12-month-old animals used in independent experiments (Figure 3E) indicated that WT mice exhibited 86.8±7.9% (mean± standard deviation [SD]) cells remaining in experimental eyes (n=5), while *Sigmar1^−/−^* mice exhibited 68.3±3.4% cells remaining (n=5) under the same conditions (p=0.0013, *t*-test). Thus, our optic nerve crush experiments demonstrated that ganglion cells in *Sigmar1^−/−^* mice underwent faster post-crush degeneration.

## Discussion

The major finding in this in vivo study is the increased susceptibility of retinal ganglion cells to optic nerve crush-induced cell death in the absence of the σR1. Because all the neurons in the ganglion cell layer were counted, of which only 40%–60% are ganglion cells [27,40,41], the maximum level of ganglion cell loss can reach only ~50% of the total counts in the control. Considering this limit, an 18.5% difference of cell loss between the WT and *Sigmar1^−/−^*is rather profound (Figure 3E). Although protective factors other than the σR1 were possibly also involved, our results nevertheless support a conclusion that the σR1 played a role in slowing down crush-induced retinal ganglion cell degeneration.

This conclusion is also supported by previous studies using various σR1 agonists. In a recent report, through intraperitoneal injection of a σR1 agonist, (+)-pentazocine, into diabetic mice, Smith et al. observed attenuated cell loss in the ganglion cell layer of retinal sections [20]. Less retinal damage has been observed also in rats following injection of other σR1 agonists, that is, PRE-084, neurosteroids, and an N-methyladamantan-1-amine derivative [(-)-MR22] [15,16,32]. Additionally, consistent evidence has been produced using primary ganglion cells [17] or RGC-5 cells [18,19], in which (+)-pentazocine mitigated cell death that was caused by the excitotoxins glutamate and homocysteine. Importantly, the current study confirms that the σR1 is a bona fide in vivo target that attenuates stress-induced retinal ganglion cell death.

It is interesting to note that under normal conditions there was no developmental difference between *Sigmar1^−/−^* and WT mice in their retinas, as indicated by essentially the same cell density in the ganglion cell layer in the *Sigmar1^−/−^* (34.41 cells/100 μm^2^) and the WT (34.02 cells/100 μm^2^). But under stress conditions exerted by optic nerve crush, a significant protective effect of the σR1 could be observed (Figure 3E). The σR1 is ubiquitously expressed in various mammalian tissues, and its sequence is highly conserved across mammalian species [1,2], as well as in zebrafish. The σR1 is thus believed to play prominent cellular and physiologic roles [2]. But it has been puzzling that there was no overt phenotype found when the *Sigmar1^−/−^*mouse model first became available [25]. It is thereby speculated that compensatory mechanisms involving the σR2 or other pathways are activated in the *Sigmar1^−/−^* mouse. Remarkably, however, some phenotypes of the *Sigmar1^−/−^* mouse could be observed when the animals were challenged by injection of σR1-binding drugs [23,25] or by forced swimming [6]. Furthermore, here we showed a phenotype of greater susceptibility of ganglion cells to degeneration in *Sigmar1^−/−^* mice when stressed by acute optic nerve crush (Figure 3E). Thus, it appears that the physiologic functions of the σR1, such as protection against ganglion cell death, are stimulated and become discernable under stressful conditions.

This stress-stimulated σR1 cellular protective action is consistent with its recently discovered role as a ligand-operated chaperone [8]. The σR1 resides in the mitochondria-associated ER membrane, and maintains mitochondrial Ca^2+^ homeostasis by chaperoning the IP3 receptor type 3 in the membrane, thus regulating its lifetime. Under various stressful conditions, the σR1 was found to translocate to the extended ER network [2,42], including the ER structure opposing the plasma membrane, whereby the σR1 is believed to be able to chaperone other proteins or regulate the functions of various ion-channels, receptors, and kinases. This probably occurs through direct interactions under some circumstances [6,8,43-48].

The molecular function of the σR1 as a chaperone/signaling modulator implies underlying mechanisms of the σR1-specific protection against the cell death observed here (Figure 3). First, the chaperoning activity of the σR1 may help contain unfolded protein responses that often lead to overproduction of the reactive oxygen species (ROS). While ROS is elevated during retinal ganglion cell degeneration [49], σR1 agonists have been found to suppress oxidative damage in the retina [20,50]. Second, since the crush-induced ganglion cell death has been previously characterized as apoptotic [33,51], the σR1 may alter gene expression in apoptosis pathways indirectly, by regulating ROS or Ca^2+^ levels. Evidence can be found from a recent report suggesting that the σR1 promoted cell survival, at least in part by transcriptionally regulating *Bcl-2* expression via the ROS/NF-kappaB pathway in Chinese hamster ovary cells [52]. *Bcl-2* and *Bax* are two important gene families in the determination of the life and death of the cell [33]. *Bcl-2* at high levels is often anti-apoptotic, whereas expression of *Bax*, up to a certain threshold, triggers cell death [53]. In another independent study, activation of the σR1 inhibited the increase of *Bax* expression in RGC-5 cells after glutamate treatments, likely by maintaining Ca^2+^ homeostasis [19]. While explanations in these studies using cultured cells are plausible, the mechanism(s) underlying the protective action of the σR1 may differ in vivo in the retinal ganglion cells, where *Bcl-x* rather than *Bcl-2* is predominantly expressed [54]. Alternatively, the σR1 may suppress the *N*-Methyl-D-aspartic acid (NMDA) currents of the ganglion cells to counter excitotoxicity [34]. Further investigations are warranted to identify the major molecular mechanism(s) of the σR1-mediated protection for the ganglion cells, preferably using *Sigmar1^−/−^* mice.

Another interesting finding in this study is the presence of the σR1 in the photoreceptor cells (Figure 2), solving a controversy that arose from previous reports [4,5]. This information raises the real possibility of using σR1-targeting therapeutic interventions to attenuate photoreceptor degeneration, which is the cause of a major blinding disease—retinitis pigmentosa [55]. Meanwhile, the same immunostaining experiments indicated a high abundance of σR1 in the ganglion cells (Figure 2). While confirming previous reports [4,5], this data also provided additional evidence supporting the cellular protective action of the σR1 (Figure 3). More importantly, abundant σR1 in mammalian retinas, as detected by both photolabeling (Figure 1) and immunohistochemistry (Figure 2), offer a valid target for pharmacological interventions. In support of in vivo σR1 regulations, several candidates of endogenous σR1 ligands have been identified, such as DMT [23], neurosteroids [22], and sphingosine [56]. Thus, activation of the σR1 using highly selective drugs may provide a practical approach to alleviating retinal neurodegeneration.

In conclusion, from our in vivo experiments using *Sigmar1^−/−^* and WT mice, and optic nerve crush surgery to induce cell death, we defined a specific role for the σR1 in containing retinal ganglion cell degeneration. Since optic nerve crush specifically triggers ganglion cell death that resembles apoptosis in glaucoma [27,28]—a major cause of blindness—the current study suggests that the σR1 may be a potential target for developing new therapies for treating this eye disease.
